# Two Homeobox Transcription Factors, Goosecoid and Ventx1.1, Oppositely Regulate Chordin Transcription in *Xenopus* Gastrula Embryos

**DOI:** 10.3390/cells12060874

**Published:** 2023-03-11

**Authors:** Vijay Kumar, Zobia Umair, Unjoo Lee, Jaebong Kim

**Affiliations:** 1 Department of Biochemistry, Institute of Cell Differentiation and Aging, College of Medicine, Hallym University, Chuncheon 24252, Republic of Korea; 2 Department of Electrical Engineering, Hallym University, Chuncheon 24252, Republic of Korea

**Keywords:** Chrd, Gsc, Ventx1.1, transcriptional regulation, organizer, *Xenopus*

## Abstract

The reciprocal inhibition between two signaling centers, the Spemann organizer (dorsal mesoderm) and ventral region (mesoderm and ectoderm), collectively regulate the overall development of vertebrate embryos. Each center expresses key homeobox transcription factors (TFs) that directly control target gene transcription. Goosecoid (Gsc) is an organizer (dorsal mesoderm)-specific TF known to induce dorsal fate and inhibit ventral/ectodermal specification. Ventx1.1 (downstream of Bmp signaling) induces the epidermal lineage and inhibits dorsal organizer-specific genes from the ventral region. Chordin (Chrd) is an organizer-specific secreted Bmp antagonist whose expression is primarily activated by Gsc. Alternatively, *chrd* expression is repressed by Bmp/Ventx1.1 in the ventral/epidermal region. However, the regulatory mechanisms underlying the transcription mediated by Gsc and Ventx1.1 remain elusive. Here, we found that the *chrd* promoter contained two cis-acting response elements that responded negatively to Ventx1.1 and positively to Gsc. In the ventral/ectodermal region, Ventx1.1 was directly bound to the Ventx1.1 response element (VRE) and inhibited *chrd* transcription. In the organizer region, Gsc was bound to the Gsc response elements (GRE) to activate *chrd* transcription. The Gsc-mediated positive response on the *chrd* promoter completely depended on another adjacent Wnt response cis-acting element (WRE), which was the TCF7 (also known as Tcf1) binding element. Site-directed mutagenesis of VRE, GRE, or WRE completely abolished the repressive or activator activity of Ventx1.1 and Gsc, respectively. The ChIP-PCR results confirmed the direct binding of Ventx1.1 and Gsc/Tcf7 to VRE and GRE/WRE, respectively. These results demonstrated that *chrd* expression is oppositely modulated by homeobox TFs, Ventx1.1, and Gsc/Tcf7 during the embryonic patterning of *Xenopus* gastrula.

## 1. Introduction

In *Xenopus*, gastrulation is a critical event that allows germ layer formation and embryonic patterning [1,2,3]. During this process, the dorsal mesoderm or organizer-specific Goosecoid (Gsc) plays an essential role in initiating inductive signaling to trigger dorso-anterior migration from the dorsal lip of the blastopore [4,5]. Alternatively, the ventral half of the embryo expresses high levels of bone morphogenetic proteins (Bmps) that target the expression of *ventx* family genes and downregulate dorsal signaling [2,6,7]. The balance between these two distinct signaling pathways drives primary dorsoventral (D-V) patterning, anteroposterior (A-P) patterning, and normal embryonic development. Several independent studies have reported that Gsc dose-dependently causes dorsalization of mesodermal tissues [4,8]. The *gsc* loss-of-function generates a headless phenotype, suggesting a crucial function in head formation [5]. Previously, it was reported that Gsc required *chrd* to enact dorsalization phenotypes, thus playing an upstream role in *chrd* activation [8,9]. Chordin (Chrd) is a secreted protein that binds to Bmp4 with a high affinity in the extracellular space. The Chrd–Bmp4 complex blocks Bmp signaling [3]. This Bmp–Chrd gradient is necessary for neuroectoderm formation and the anteroposterior axis in vertebrates [3]. In combination, Gsc induces the neuronal program and head formation via two routes. First, it inhibits the transcription of the neural repressor (ventral; Bmp/Ventx1.1 signaling), and second, it activates *chrd* expression to minimize Bmp signals [5,8,9]. However, the detailed molecular mechanism has not been fully elucidated; therefore, we investigated the functional role of Gsc in activating *chrd* transcription. In contrast, Bmp signaling is predominant in the ventral region of the early gastrula embryo, promoting ectodermal differentiation [10,11]. We reported that Bmp signaling activates the expression of Ventx1.1 (a homeobox transcription factor [TF]) [12] to repress the expression of organizer and neural target genes [6,13,14]. The Ventx1.1 gain-of-function analyses indicate that Ventx1.1 produces the headless phenotype by blocking the manifestation of organizer and neural target genes [2,6,13]. Furthermore, a study of various truncated proteins revealed that the active repressor activity is mediated by the C-terminal domain of Ventx1.1 [14]. The ectopic expression of the Ventx1.1 fusion protein (fused with engrailed domain; a transcription repressor) shows antimorphic effects that allow the expansion of organizer-specific genes, resulting in the partial secondary axis in the *Xenopus* tadpole [15], suggesting the requirement of Ventx1.1 for normal development.

In the present study, we hypothesized that *chrd* is a common downstream target gene of Bmp/Ventx1.1 and Gsc during the embryonic development of *Xenopus*. We aimed to investigate the opposite regulatory mechanism of *chrd* transcription via the two organizer- and ectoderm-specific homeobox TFs, Gsc and Ventx1.1, in gastrula embryos.

## 2. Materials and Methods

### 2.1. Ethics Statement

Animal studies were conducted according to the Institutional Animal Care and Use Committee regulations of Hallym University (Hallym 2021-91, 2021-92, 2019-79). All research team members attended educational and training courses for the appropriate care and use of experimental animals. Adult *Xenopus laevis* were maintained in suitable containers under a 12 h light/dark (LD 12:12 h) cycle at 18 °C by authorized personnel for laboratory animal maintenance according to the Institute of Laboratory Animal Guidelines Resources of Hallym University.

### 2.2. DNA and RNA Preparation

*Flag-Ventx1.1, Flag-Gsc, and Myc-Tcf7* mRNAs used in the study were linearized using NotI/SacII restriction enzymes. The linearized vectors (pCS4-Flag-Ventx1.1, pCS4-Flag-Gsc, and pCS4-Flag-Tcf7) were used for in vitro transcription assays using the MEGA script kit (Ambion, Austin, TX, USA) following the manufacturer’s instructions [16]. Synthetic mRNAs were then quantified at 260/280 nm using a spectrophotometer (SpectraMax, Molecular Devices, San Jose, CA, USA) and diluted in DEPC water to a final concentration of 1 ng/5 nL. A 5 nL solution was injected into each embryo to deliver 1 ng mRNA.

### 2.3. Cloning of Chrd Genomic DNA

**Figure 1:**
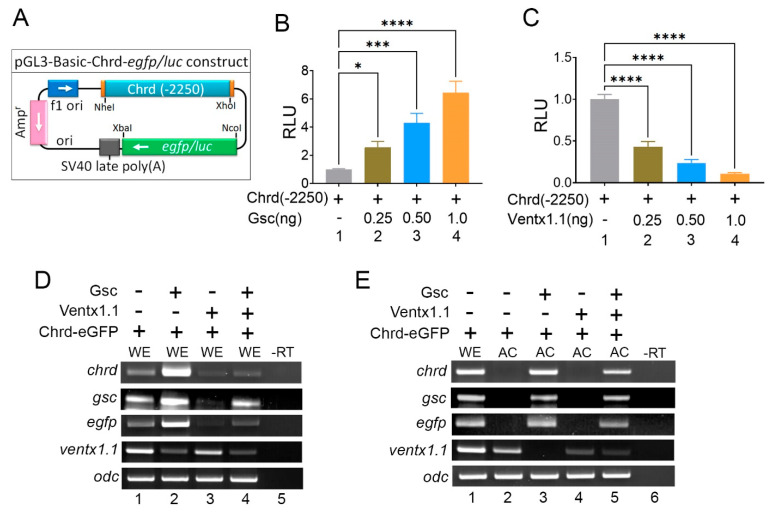
Ectopic expression of Gsc and Ventx1.1 differentially regulates the dorsal mesoderm (organizer) gene expression during gastrula. (**A**) The cloned *chrd(*-*2250)* promoter mapped into Pgl3 luc/eGFP vector. (**B**,**C**) The *Xenopus* embryos were injected with Gsc (**B**) and Ventx1.1 (**C**) (1 ng/embryo) at the one-cell stage. The reporter assay was performed at stage 11 in whole embryos (WEs). (**C**,**D**) To examine the endogenous expression, embryos were co-injected with Gsc and Ventx1.1 (1 ng/embryo) at the one-cell stage. RT-PCR was performed at stage 11 in Wes (**D**) and ACs (**E**). -RT referred to control reaction without reverse transcriptase.

Cloning of the *chrd* promoter region (−1 to −2250 bp) from genomic DNA (gDNA) (Xenbase gbrowse laevis 9.1: chr5S:81716980-81719233) was performed using specific primers as previously described [16]. First, the amplified DNA product was cloned into the pGL3-Basic plasmid (Promega, Madison, WI, USA) using NheI/XhoI (Promega) restriction enzymes; this construct was referred to as “*chrd(-2250)luc*” (Figure 1A).

### 2.4. Chrd Promoter Constructs

The *chrd(*-*2250)luc* construct was used to design serially deleted promoter constructs, as shown in Figure 1A, and the primers used are listed in Table 1. The cloning methods for *chrd(*-*2250)luc* and *chrd(*-*2250)eGFP* constructs were similar to those reported previously (Figure 1A) [16,17].

### 2.5. Embryo Injection and Explant Culture

*X. laevis* females were injected with 500 units of human chorionic gonadotropin hormone (Sigma, St. Louis, MO, USA) to obtain oocytes. The obtained oocytes were subsequently fertilized in vitro, and embryos were injected into the animal pole at the one-cell stage with DNAs/RNAs as previously described [16,17]. Animal cap explants (ACs) were dissected from the injected/non-injected embryos at the blastula stage (stage 8). The dissected ACs and whole embryos were cultured in 1× L-15 medium (Gibco/Thermo Fisher, Waltham, MA, USA) and 30% Marc’s Modified Ringer (MMR) solution until they reached stage 11/11.5. 

### 2.6. RT-PCR

*Flag-Ventx1.1 and Flag-Gsc* mRNA (1 ng/embryo) were injected into the animal pole at the one-cell stage. Embryos were cultured in 30% MMR solution. ACs were subsequently dissected from the injected and non-injected embryos at stage 8 and incubated in 1 × L-15 growth medium until they reached stage 11, as previously described [18]. Total RNA was isolated from whole embryos and ACs using TRIzol reagent (Ambion, USA) according to the manufacturer’s instructions. The isolated RNA samples were treated with DNase I to remove contaminating DNA. As per the manufacturer’s instructions, the cDNA was prepared with 1 μg of total RNA per reaction using R32301, HiScript III RT SuperMix (Vazyme, Nanjing, China). Thermal cycling was performed as follows: 30 s at 95 °C, 30 s at each annealing temperature, 30 s at 72 °C, and 20–30 cycles of amplification (Table 2).

### 2.7. Luciferase Assays

The serially deleted and mutant constructs of *chrd(*-*2250)luc* were injected with or without Ventx1.1, Gsc, or Tcf7 mRNAs, and reporter assays were performed as previously described [13]. Relative promoter activity was measured using a luciferase assay system (Promega) according to the manufacturer’s instructions for the reporter activity assay. Five sets of embryos (three embryos/group) were harvested at stage 11/11.5 and homogenized in 10 μL of lysis buffer/embryo. Embryo homogenates (10 μL each) were assayed with 40 μL of luciferase substrate, and reporter gene activity was measured using an illuminometer (Berthold Technologies, Bad Wildbad, Germany). All experiments were performed separately, in triplicates (minimum).

### 2.8. Site-Directed Mutagenesis

Point mutations were generated using a site-directed mutagenesis kit (Muta-Direct, iNtRON Biotechnology, Seongnam, Republic of Korea) with specific primers (Table 3), according to the manufacturer’s instructions and as previously described [19]. 

### 2.9. Chromatin Immunoprecipitation (ChIP)

The ChIP assay was performed as previously described [20]. mRNAs encoding Flag-Ventx1.1, Flag-Gsc, and Myc-Tcf7 (1 ng/embryo) were injected at the one-cell stage. Injected embryos were harvested at stage 11 (80–90 embryos/sample) and handled according to the protocol. Anti-Flag/Myc polyclonal antibody (Santa Cruz Biotechnology, Dallas, TX, USA) or normal mouse IgG (SC-2025, Santa Cruz Biotechnology, Dallas, TX, USA) were subsequently added to the cell lysates to immunoprecipitate the chromatin. Finally, ChIP-PCR was performed with immunoprecipitated chromatin using specific primers for the Ventx1.1 response element (VRE), Gsc response element (GRE), and Wnt response element (WRE). As previously shown, a random primer set was designed to test ChIP efficiency, i.e., the internal negative control (C) [17]. The primers used are listed in Table 4.

### 2.10. ChIP-Sequencing Analysis

The Ventx1.1 mRNA (1 ng/embryo) was injected at the one-cell stage, and embryos (approximately 1000 were used) were harvested at stage 11. The ChIP assay was performed as previously described [20]. Immunoprecipitated total chromatin was sequenced by Macrogen (Seoul, Republic of Korea), and raw data (short reads) were obtained in the FASTA format. The Galaxy (https://usegalaxy.org accessed on 15 June 2020) online tool was used for data analyses [21]. Finally, MACS call peak data were visualized, and Ventx1.1 coverage was plotted for the *chrd* promoter region shown in Figure 3C.

### 2.11. eGFP Fluorescence

The *chrd(-2250)eGFP* (indicated as chrd-eGFP) construct was injected (200 pg/embryo) with or without Gsc and Ventx1.1 mRNA (1 ng/embryo) at the one-cell stage and into the animal hemispheres separately. eGFP fluorescence was assessed using a stereo microscope with a royal blue light adapter (Stereo Microscope Fluorescence Adapter, NIGHTSEA, Lexington, MA, USA), and images were captured using a Nikon D810 camera (Nikon, Tokyo, Japan) as described previously [16,17].

### 2.12. Statistical Analyses

Data were analyzed using unpaired two-tailed Student’s *t*-test or a one-way analysis of variance (ANOVA) using GraphPad Prism 9.4 (GraphPad, San Diego, CA, USA). Significance values were set as * for *p* ≤ 0.1, *** for *p* ≤ 0.001, and **** for *p* ≤ 0.0001; n.s. denotes nonsignificant values.

## 3. Results

### 3.1. Gsc and Ventx1.1 Oppositely Regulate Chrd Expression

Gsc is a signature TF of the organizer that executes its function in embryonic dorsal mesoderm and neuroectoderm formation and is also capable of generating a secondary axis when overexpressed [8,9,22]. Previous reports have confirmed that Gsc activates *chrd* expression to deploy dorsalizing effects [8,22]. Alternatively, Bmp4 signaling induces the expression of Ventx1.1, a well-known repressor that strongly inhibits organizer genes, including *chrd* and neural-specific genes, to protect the ectodermal fate [6]. Overexpressed *ventx1.1* ventralizes embryos or generates headless phenotypes [13,14]. Thus, Gsc and Ventx1.1 were expected to be exclusively present in the dorsal and ventral regions of the embryo to modulate opposing organizer genes, including *chrd*. To investigate the molecular mechanism, the -2250 bp long *chrd* promoter (*chrd*(-*2250*)) from the *chrd* translation start site, (TLS) was cloned into the pGL3-reporter vector (Figure 1A). The cloned promoter *chrd(-2250)* contains activin and Foxd4l1.1 response elements [16,17]. To examine the reporter activity, *chrd(-2250)* was co-injected with or without Gsc and Ventx1.1 mRNA at the one-cell stage of *Xenopus* embryos. The results showed that Gsc increased the reporter gene activity in a dose-dependent manner (Figure 1B, first to fourth bars). In contrast, Ventx1.1 gradually decreased the reporter activity (Figure 1C, first to fourth bars). We examined the effects of *gsc* (1 ng/embryo) and *ventx1.1* (1 ng/embryo) on the endogenous *chrd* expression in both whole embryos (WEs) and animal cap explants. *eGFP* expression was also evaluated to examine whether the cloned eGFP reporter construct (*chrd(-2250)eGFP)* expressed the *egfp* transcript similar to that of the endogenous *chrd*. RT-PCR results showed that Gsc induced *chrd, gsc*, and *egfp* expression while inhibiting the *ventx1.1* expression at stage 11 in WEs (Figure 1D, lanes one to four) and ACs (Figure 1E, lanes two to five). Alternatively, Ventx1.1 inhibited *chrd* and *egfp* expression in WEs and ACs (Figure 1E, lanes two to five). The co-injection sample of *gsc* and *ventx1.1* showed a similar level of *chrd* expression to that of the uninjected WEs (Figure 1D, lane four) and a marginal increase in *chrd* and *egfp* expression in ACs (Figure 1E, lane five). The results showed that two homeobox TFDs, Gsc and Ventx1.1, oppositely modulated the *chrd* and *chrd(-2250)* reporter activity, indicating that the isolated promoter contains the expected cis-acting elements of Gsc and Ventx1.1.

### 3.2. Chrd(-2250)eGFP Reporter Shows Positive and Negative Responses to Gsc and Ventx1.1 mRNAs, Respectively, in Gastrula Whole Embryos

**Figure 2:**
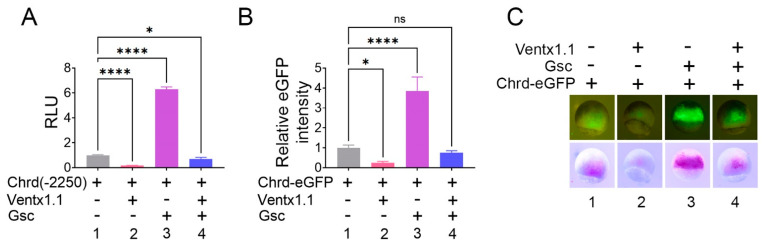
The *chrd(-2250)* promoter region contains the positive and negative response elements for Gsc and Ventx1.1., respectively. (**A**) *chrd(-2250)* was injected with or without *gsc* (1 ng/embryo) and *ventx1.1* (1 ng/embryo) at the one-cell stage. The luciferase reporter gene assay was performed at stage 11. (**B**) The quantification of eGFP relative fluorescence was analyzed with the embryos shown in (**C**). The *chrd(-2250)eGFP* was injected with or without *gsc* (1 ng/embryo) and *ventx1.1* (1 ng/embryo) at the one-cell stage, and fluorescent analysis was performed at stage 10.5. ). * *p* ≤ 0.1, **** *p* ≤ 0.0001, and n.s. denotes nonsignificant values.

*gsc* and *ventx1.1* mRNAs modulated the endogenous *chrd* expression and isolated the -2250 bp promoter (Figure 1D,E). Thereafter, we examined the *chrd(-2250)eGFP* construct to visualize the response in gastrula embryos. The reporter activities of both *chrd(-2250)luc and eGFP* significantly increased by co-injection with *gsc* (1 ng/embryo, about 6-fold), wherein *ventx1.1* injected embryos that showed a markedly reduced expression (0.2-fold) (Figure 2A, 1 to 3). However, double (*gsc* and *ventx1.1*)-injected embryos did not show any significant changes in either *chrd(-2250)luc* or *eGFP* reporter gene expression (Figure 2A, 1 vs. 4), as shown in Figure 1D. As shown in Figure 2C, eGFP fluorescence was observed in *Xenopus* gastrula embryos. As expected, the fluorescence intensity markedly increased (approximately 4-fold) in *gsc-*injected embryos compared with that in the control embryos (Figure 2C, first left panel). Alternatively, *ventx1.1* reduced the fluorescence intensity (Figure 2C, second left panel, 0.25-fold). The co-injection of *gsc* and *ventx1.1* led to embryos showing a fluorescence intensity similar to that of uninjected embryos (Figure 2C, fourth panel), as shown in Figure 1D, lane four. Together, these observations suggested that the *chrd(-2250)* promoter contains *cis*-acting response element(s) for Gsc and Ventx1.1 within the isolated promoter region.

### 3.3. Chrd Promoter Contains VRE

**Figure 3:**
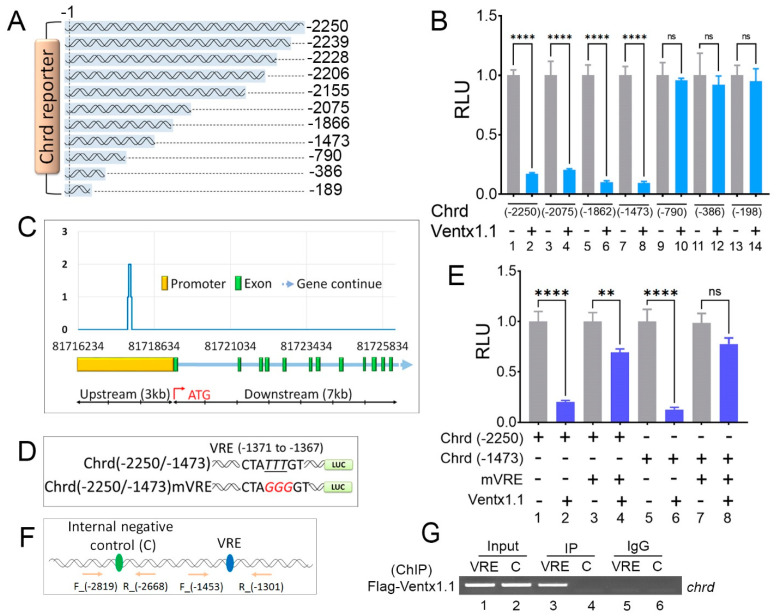
*chrd* promoter contains VRE. (**A**) Schematic representation of serially deleted *chrd* promoter constructs. (**B**) Relative luciferase activity of serially deleted *chrd* promoter constructs with or without Ventx1.1. (**C**) ChIP-sequencing coverage plot of Ventx1.1 within the *chrd* promoter region. (**D**) Schematic representations of site-directed mutagenesis and targeted nucleotides are italicized, underlined, and shown in red. (**E**) Relative luciferase activities of *chrd(-2250), chrd(-2250)mVRE, chrd(-1473),* and *chrd(-1473)mVRE,* with or without Ventx1.1. (**F**) The location of designed primers for VRE and the internal negative control (**C**). (**G**) Chromatin immunoprecipitation (ChIP) assay was performed to test the occupancy of Ventx1.1-Flag to VRE within the promoter region of *chrd*. ** *p* ≤ 0.01, **** *p* ≤ 0.0001, and n.s. denotes nonsignificant values.

Several serially deleted promoter constructs were cloned to identify functionally active VRE within the *chrd(-2250)* promoter (Figure 3A). The serially deleted constructs were injected with or without *ventx1.1,* and the luciferase activity was measured in the injected embryos. The reporter assay results showed that the co-injection of ectopic *ventx.1.1* significantly reduced the reporter activities of the *chrd(*-*2250)*, *(*-*2075)*, *(*-*1862)*, and *(*-*1473)* constructs (Figure 3B, bars one to eight). The constructs containing shorter than -*1473* bp, including *chrd(-790)*, *(-386)*, and *(-198),* showed that the reporter activities did not respond to Ventx1.1 (Figure 3B, bars nine to fourteen). The results indicated that the putative VRE resides between the -1473 and -790 bp regions of the *chrd* promoter. We previously performed genome-wide ChIP-sequencing (ChIP-seq) of Ventx1.1 [13]. Notably, the ChIP-seq coverage plot of the *chrd* genomic DNA region showed one specific coverage peak in the −1439 to −1305 bp region within the *chrd* promoter (Figure 3C). In addition, the −1439 to −1305 bp promoter region contains the putative Ventx1.1 homeobox binding consensus sequence found at the −1371 to −1367 bp (TATTTG) region of the *chrd* promoter [13,23]. Site-directed mutagenesis was performed to mutate the three conserved nucleotides within VRE (TA*TTT*G) into mutated VRE (mVRE; TA*GGG*G). Two mutated reporter constructs, *chrd(-2250)mVRE* and *chrd(-1473)mVRE*, were cloned (Figure 3D). The reporter activity was measured after co-injection with or without *ventx1.1*. The reporter activities of *chrd(-2250)mVRE* and *chrd(-1473)mVRE* showed strong attenuation of the negative response observed in the wild-type promoter constructs (Figure 3E). However, the reporter activity of *chrd(-2250)mVRE* in the presence of *ventx1.1* did not recover to that of *chrd(-2250)* (in the absence of *ventx1.1*) (Figure 3E, compare third and fourth bars). We reasoned that the ectopic expression of Ventx1.1 can also inhibit *chrd* activators such as *gsc* expression (Figure 1), which will be discussed in the following Discussion section. To confirm the direct binding of Ventx1.1 to the VRE region on the endogenous *chrd* promoter, ChIP-PCR was performed. As shown in Figure 3F, a PCR band containing the VRE region was detected in the IP sample of the ChIP PCR assay (Figure 3G, third lane). Collectively, these results suggested that Bmp4 targets the TF Ventx1.1, which directly binds to the VRE region at -1371 to -1367 bp (TATTTG) of the *chrd* promoter to repress the *chrd* transcription. 

### 3.4. Gsc Requires Both GRE and WRE Cis-Acting Response Elements to Activate Chrd Transcription

**Figure 4:**
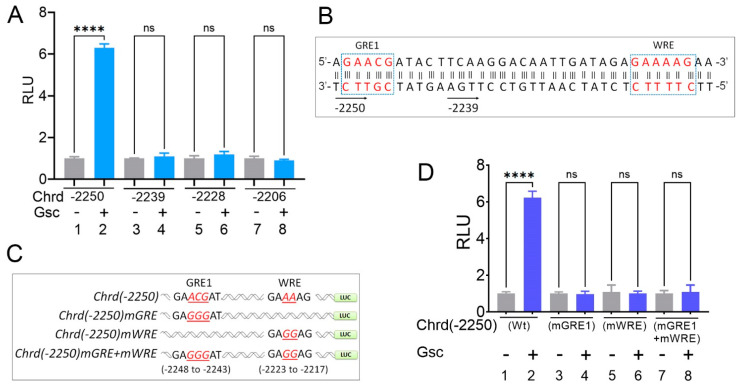
Site-directed mutagenesis of GRE1 and WRE within the *chrd* promoter completely abolishes Gsc-mediated transcriptional activation. (**A**) Relative luciferase activity of serially deleted *chrd* promoter constructs with or without Gsc. (**B**) *Chrd* promoter (-2250 to -2214 bps)with highlighted GRE1 and WRE (dotted boxes). The bottom arrowhead indicates the sequences of *chrd(-2250)* and *chrd(-2239)* reporter constructs. (**C**) Schematic representation of the site-directed mutagenesis scheme; targeted nucleotides are shown in italics and red. (**D**) Relative luciferase activity of *chrd(-2250)mGRE1* and *chrd(-2250)mWRE* constructs with or without Gsc. **** *p* ≤ 0.0001, and n.s. denotes nonsignificant values.

Consistent with previous reports, we found that ectopic Gsc strongly induced the endogenous chrd expression and chrd reporter activities (Figure 1 and Figure 2) [2,8,9,24]. To identify the GRE within the *chrd(-2250)* promoter, serially deleted constructs were cloned (Figure 3A). The serially deleted constructs were injected with or without *gsc* to measure the reporter activity with the injected embryos. The reporter assay results showed that the co-injection of ectopic *gsc* significantly increased the reporter activity of the longest *chrd(-2250)* promoter construct (approximately 6.2-fold) (Figure 4A). All shorter promoter constructs, including *chrd(-2239)* to *chrd(-198)*, showed no positive response to the injected *gsc* (Figure 4A and Figure S1). Only an 11 bp shorter construct of *chrd(-2239)* compared to *chrd(-2250*) did not show any positive response to *gsc,* suggesting that the putative GRE resides within the upstream 11 nucleotides (from −2250 to −2239). To determine the exact response to nucleotide sequences, a site-directed mutagenesis was performed to generate two different point mutations within the upstream 11 nucleotides, which were termed *chrd(-2250)mGRE1* and *mGRE2* (Figure S2A). The three nucleotides (GA*ACG*ATACTT to GA*GTA*ATACTT) were replaced in the first mutant *(mGRE1)* and the last three nucleotides (GAACGATA*CTT* to GAACGATA*AGG*) within the targeted 11 nucleotides were in the second mutant *(mGRE2),* as shown in Figure S2A,B, respectively (the mutated nucleotides are shown in *italics* and are underlined). The *chrd(-2250)mGRE1* and *mGRE2* constructs were then injected with or without *gsc* to examine the reporter activity. The reporter gene assay showed that the positive response was completely abolished in *chrd(-2250)mGRE1.* However, *chrd(-2250)mGRE2* showed the same positive response to *gsc* as the wild-type *chrd(-2250)* (Figure S2C). The results suggested that the organizer-specific TF, Gsc, requires GRE1 to activate the *chrd* transcription. Gsc is reported to be a transcription repressor that inhibits Xwnt8-mediated signaling to promote the head organizer [22]. Our recent study also supports the use of Gsc as a repressor. Gsc directly represses the expression of early neural genes, including *foxd4l1.1* and *zic3,* in the mesodermal region [9]. Alternatively, Gsc induces the expression of secreted protein factors, *chrd* and *noggin* (potent BMP inhibitors), to convert the proximal ectoderm to neuroectoderm in Bmp-inhibited conditions in the same report [9]. Therefore, Gsc may function either as a repressor or an activator in a context-dependent manner, and it requires an additional co-activator working with Gsc to activate *chrd* transcription. Notably, the conserved consensus sequence (AAAG), also known as Tcf7 binding *cis*-regulatory motifs (WWCAAAG or CTTTG(A/T)(A/T)), which functions downstream of Wnt signaling [25,26], was found in a proximity region (−2223 to −2217) to active GRE1 (−2250 to −2246 bp). We hypothesized that a putative WRE (Figure 4B) is also involved in the *chrd* transcriptional regulation. Site-directed mutagenesis of *chrd*(-2250)*mWRE* was performed using two conserved nucleotides (AAAG to *GG*AG) at the four conserved consensus sequences (AAAG), as shown in Figure 4C. Notably, the results showed that the Gsc-mediated positive response was completely lost in mutated *chrd*(-2250)*mWRE* alone or in double-mutated *chrd*(-2250)*mGRE1*+*mWRE* (Figure 4D, bars one to eight). Note that *chrd(-2239)*, which did not respond to Gsc yet contained the WRE, indicated that the intact positive response of Gsc on *chrd* transcription is not possible without either cis-acting GRE or WRE. These results suggested that cis-acting elements of both intact GRE1 and WRE are required for Gsc-mediated *chrd* transcriptional activation. 

### 3.5. Tcf7 Directly Binds on the WRE Region to Activate Chrd Transcription

**Figure 5:**
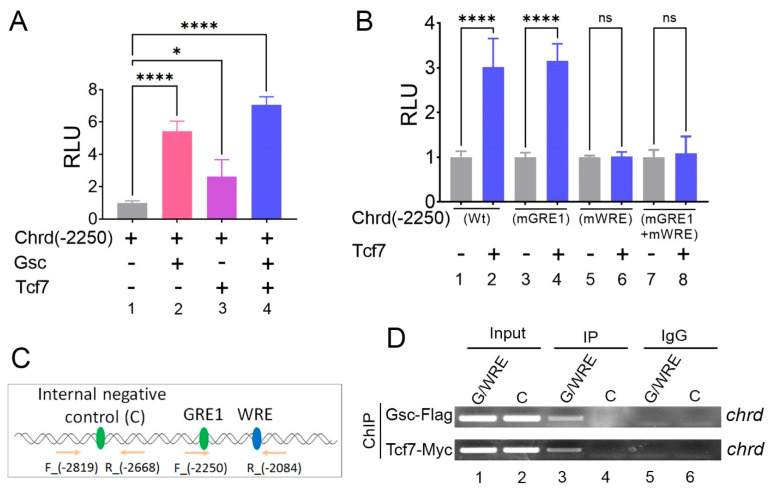
Tcf7 binds to WRE to activate *chrd* transcription. (**A**) Relative luciferase activity of *chrd(-2250)* promoter constructs with Gsc and Tcf7. (**B**) Relative luciferase activity of *chrd(-2250)mGRE1* and *chrd(-2250)mWRE* constructs with Tcf7. (**C**) Schematic representation of designing the ChIP-PCR primer for GRE, WRE, and the internal negative control. (**D**) Chromatin immunoprecipitation (ChIP) assay was performed to test the occupancy of Gsc-Flag and Tcf7-Myc to GRE/WRE within the promoter region of *chrd*. * *p* ≤ 0.1, **** *p* ≤ 0.0001, and n.s. denotes nonsignificant values.

We found that the WRE located in the proximal region of GRE1 within the *chrd(-2250)* promoter was critical for the Gsc-mediated activation of *chrd* transcription. Previous studies have reported that Tcf7 induces *chrd* expression in *Xenopus* embryos [27,28]. Additionally, a recent study demonstrated that the active Wnt/β-catenin signaling-mediated induction of organizer genes requires two steps [26]. First, at a low level of nuclear β-catenin, Tcf7l1 forms a complex with Groucho (co-repressor) and inhibits Wnt target genes. In the second step, increased nuclear β-catenin switches the Tcf7l1 complex to lymphoid enhancer-binding factor 1 (Lef1), a transcription activator, to activate Wnt target genes [26]. As the presence of WRE on the *chrd* promoter suggests the possibility of Wnt/Tcf involvement, we examined Tcf7 involvement with *chrd(-2250)*. The *chrd(-2250)* reporter construct was injected with or without *tcf7* and *gsc* mRNA. The luciferase results showed that Tcf7 alone increased reporter gene activity approximately 2.5-fold (Figure 5A, first vs. third bars). Co-injection of *tcf7* and *gsc* enhanced reporter activity (approximately 6- to 7-fold) more than that of *tcf7 or gsc* injection alone (Figure 5A, bars two to four). To further evaluate the effect of Tcf on the *chrd* promoter, *chrd(-2250)mWRE* or *chrd(-2250)mGRE1* reporter gene constructs were injected with or without *tcf7* mRNA. Both constructs of WRE site-mutated *chrd* promoter, *chrd(-2250)mWRE* and *chrd(-2250)mGRE1*+ *mWRE,* lost the positive response to Tcf7, which was shown in *chrd(-2250)* (Figure 5B, fifth to eighth bar). In contrast, mutations in GRE1 (*mGRE1*) did not attenuate the Tcf7-mediated positive response. Both constructs of *chrd(-2250)* and *chrd(-2250)mGRE* showed similar reporter activity, as shown in Figure 5B (second vs. fourth bar). These results suggested that Tcf7-mediated *chrd* transcription activation is dependent on intact WRE and not on the presence of intact GRE, which is different from the Gsc results, in which both cis-acting elements of intact *GRE1* and *WRE* were required. ChIP-PCR was performed to confirm the direct binding of Gsc and Tcf7 to the endogenous *chrd* promoter. PCR bands containing the *GRE1/WRE* region of the *chrd* promoter were detected with the IP of both Gsc- and Tcf7-injected samples (Figure 5C,D). The ChIP-PCR results suggested that Gsc and Tcf7 directly bind to GRE/WRE within the *chrd* promoter. Altogether, Gsc-mediated activation required both elements of intact GRE and WRE, and Tcf7 required only WRE for *chrd* transcription.

## 4. Discussion

In the present study, we aimed to address how an organizer-specific gene is regulated in different embryonic regions. We focused on the central signal of the ventral region (Bmp/Ventx1.1) and the antagonist signal (Gsc) from the dorsal center. In the ventral region, the BMP signal dominantly maintains the ventral ectoderm or mesoderm characteristics and inhibits the dorsal (neuroectoderm) or mesoderm (organizer). In the organizer region, Bmp antagonists, including *chrd*, are emanated to inhibit the Bmp signal and protect it from the ventral ectoderm (epidermis). Based on the existing evidence, we selected *chrd* as a common target gene that was negatively regulated by Bmp in the ventral region and positively regulated by activin/nodal in the dorsal mesoderm during gastrulation. Bmp signaling is highly conserved throughout the vertebrates and it plays a crucial regulatory role in defining the embryonic patterning [6,29,30]. The present study was designed to elucidate the mechanism underlying the Bmp-mediated transcriptional regulation of *chrd.* Additionally, we selected Gsc as a signature TF of the dorsal region and well-known TF under activin/nodal signaling, which has been reported to activate *chrd* transcription in *Xenopus* gastrula [5,8,22,31].

### 4.1. Ventx1.1 Inhibits Chrd Transcription to Protect Ectoderm Fate

During gastrulation, BMP signaling transcriptionally activates the *ventx* family TFs, including Ventx1.1, Ventx2.1, and Ventx3.2, which drive ectoderm/epidermal specification [6]. Here, we selected Ventx1.1 as a target TF to inhibit the *chrd* transcription, preventing non-ectodermal fate during gastrulation. The reasons for selecting Ventx1.1 as a *chrd* repressor are discussed. First, Ventx1.1 plays an important role in epidermal differentiation by inhibiting the expression of non-ectodermal genes in the ectodermal region. Previous studies have demonstrated that ectopic *ventx1.1* significantly reduces or abolishes the expression of mesodermal *gsc*, *chrd*, *xbra*, and *follistatin* and neural-specific *zic3* and *foxd4l1.1* [8,12,13,18,23,32]. Additionally, *ventx1.1* mRNA injected in the dorsal region of four-cell stage *Xenopus* embryos produces the headless phenotype, suggesting a dominant role in the preoccupied embryonic territory [15,32]. Ventx1.1 also represses the activation of organizer target genes, including *chrd, noggin (nog),* and *gsc* [6,13,15]; however, the exact mechanism remains unknown. Second, our genome-wide ChIP-sequencing revealed that Ventx1.1 occupied the 5′ flanking region of the *chrd* promoter. Third, the expression of the *chrd(-2250)* reporter construct was markedly reduced by the ectopic *ventx1.1 mRNA*. Conversely, morpholino-based knockdown of the *ventx1.1* gene causes dorsalized phenotypes in *Xenopus* embryos [6], indicating that *ventx1.1* depletion expands dorsal signaling and reverts ventral fate. These findings suggest the inhibitory regulation of Ventx1.1 on non-ectodermal genes to sustain ectodermal lineages during early development. Previous studies have shown that Bmp/Smad1 activates *ventx* family genes, including *ventx1.1*, *ventx1.2*, *ventx2.1*, and *msx1*, collectively inhibiting organizer- and neural-specific genes to promote epidermis formation [3,10,13,23,32].

Here, we demonstrated that ectopic expression of *ventx1.1* inhibits reporter gene expression under the *chrd(-2250)* promoter in a dose-dependent manner. Similarly, Ventx1.1 also inhibits the endogenous expression of organizer-specific genes *chrd* and *gsc* gene expression in whole embryos and animal cap systems. We noticed a similar expression pattern for the *chrd(-2250)eGFP* reporter gene to that for the endogenous *chrd.* These results suggested that Ventx1.1 strongly inhibits the expression of *chrd* in *Xenopus* embryos. These results are consistent with previous studies demonstrating the conserved repressor activity of Ventx1.1 for non-ectodermal genes in other vertebrate models [6,13,33,34]. These findings collectively indicate that Bmp/Ventx1.1 plays an important inhibitory role on germ layer-specific factors necessary for normal dorsoventral patterning. Furthermore, we explored the genome-wide target of Ventx1.1 at the gastrula stage, as the whole genome of *Xenopus laevis* has been sequenced [35]. The genome-wide ChIP-sequencing data revealed that Ventx1.1 occupied (from −1439 to −1305) the 5′ flanking region of the *chrd* promoter. Our ChIP-sequencing data showed that Ventx1.1 also occupied the *gsc* promoter region (data not shown), indicating that *chrd* and *gsc* may be direct targets of Ventx1.1. The inhibition of *chrd* by Ventx1.1 could be due to the presence of conserved cis-acting elements (VRE; TATTTG) in the promoter region [13,23]. To investigate this possibility, a point mutation in VRE (TA*TTT*G to TA*GGG*G) was generated via site-directed mutagenesis, which significantly restores the Ventx1.1 repressor activity. Further ChIP-PCR results confirmed the binding of Ventx1.1 to the VRE region within the *chrd* promoter. These results suggested that in the ectoderm, Bmp/Smad1 activates Ventx1.1, which binds to the VRE within the *chrd* promoter to inhibit transcription. We have shown the precise molecular mechanism explaining that Ventx1.1-mediated regulation of *chrd* transcription may be necessary to sustain ectodermal lineage specification in *Xenopus* embryos. 

### 4.2. In the Organizer Region, Gsc Activates Chrd Transcription to Induce Embryonic Patterning

Gsc plays a crucial role in embryonic patterning by promoting the dorsal fate (dorsal mesoderm or organizer) [22,36,37,38]. Ectopic Gsc expression impaired normal development, allowing the expansion of the dorsal tissue, whereas depletion caused a reduction in the head structure, cyclopia, and enlarged ventral tissues. It is accepted that Gsc activates *chrd* transcription to induce embryonic patterning [8]. A recent study demonstrated that Gsc also required Chrd to activate the neuronal program and neural patterning in a Bmp-inhibited manner [9]. Thus, Gsc plays an upstream role in activating *chrd* transcription in the dorsal mesoderm (organizer) [8,9]; however, its regulatory mechanism remains unknown. In the present study, we addressed the question of how Gsc activates *chrd* at the transcription level in *Xenopus* embryos. To answer this question, we checked the endogenous *chrd* and reporter gene expression. The results indicated that Gsc robustly induced both endogenous *chrd* expression and reporter gene activity. Consistent with previous reports, Gsc significantly reduced the expression of non-organizer genes, such as the Bmp target *ventx1.1* [8,9,24]. Thus, we further investigated the response elements (GRE) within the *chrd(-2250)* promoter. Collectively, the results from reporter gene assays of serially deleted *chrd(-2250)* constructs and site-directed mutagenesis suggest that GRE1 is a functionally active *cis-*acting element for the Gsc-mediated activation of *chrd* transcription. Finding GRE within *the chrd* promoter is fascinating because it is a well-known transcription repressor [22]. The mechanism of *chrd* activation by Gsc remains to be addressed; however, two possible answers have been proposed. First, Gsc represses the dorsal antagonist Bmp/ventx pathway that promotes dorsal signaling; for example, Bmp downstream of Ventx1/2 inhibits *gsc*, *chrd*, *nog*, and other organizer and neural target genes [8,13,15,23,32,34]. Thus, inhibiting Bmp and target genes allows the expression of counterpart genes in *Xenopus* embryos [2,6,8]. Additionally, Gsc inhibits Wnt8 signaling to trigger head organizers [22,31]. The second possibility is that Gsc may require co-activators to deploy activator activity [26]. A previous study showed that Wwp2 (E3 ubiquitin ligase) interacts with Gsc and facilitates mono-ubiquitylation, and ubiquitylated Gsc (Ub-Gsc) acts as a transcriptional activator. The optional transcriptional activator role of Ub-Gsc is an essential step in switching the *sox6* gene for craniofacial development in mice [39]. It should be noted that *Chrd–/–* and *Nog+/–* mice exhibit developmental defects in the head region, such as cyclopia and holoprosencephaly [40]. Additionally, nonfunctional mutations in the *chrd* gene generate head-related congenital malformations [41]. These reports recapitulate the possibility of the Gsc-mediated activation of *chrd, nog,* and *sox6* during vertebrate embryonic development [8,31,39,40,41]. The presence of GRE within the *chrd* promoter suggests that Gsc may act as an independent activator or interact with some co-activators to switch on *chrd* transcription. However, how Gsc activates *chrd*, whether through a post-translational modification that gives activator activity such as, is an interesting subject for future investigation [39]. There is a high possibility of interaction between some activators or co-activators with Gsc in this process. We did not explore this possibility in the present study.

### 4.3. Chrd Promoter Contains WRE for Tcf7-Mediated Transcription Activation

Several studies have demonstrated that Wnt/β-catenin signaling plays an important role in the organizer formation by activating the target genes [26,42,43,44]. Previous studies have reported conserved Wnt/Tcf cis-acting elements in vertebrates (WWC*AAAG* or CTTTG(A/T)(A/T) [25,26] and invertebrates (A/T(T)C/T(*AAAG*)) [43,45]. Notably, the *chrd* promoter contained four conserved nucleotides (GA*AAAG*) (italicized and underlined) in proximity to GRE1. Interestingly, a point mutation in either GRE1 or WRE completely abolished the Gsc-mediated *chrd* activation. As several studies have shown, we sought to test Tcf7 using the *chrd(-2250)* reporter gene [26,43,45,46]. Notably, Tcf7 also induced a 2.5-fold increase in the reporter gene expression. These results support those of previous reports, wherein ectopic Tcf7 robustly induces *chrd* expression in *Xenopus* [28]. However, different Tcf7 isoforms show functional differences in activating organizer-specific genes and producing distinct *Xenopus* phenotypes [27]. We observed that Tcf7 recognizes only the WRE site, suggesting that Wnt/Tcf signaling activates organizer-specific genes, such as *chrd,* by binding to conversed cis-acting elements [25,26,27]. N-terminal truncated Tcf3 (ΔN-Tcf3) completely abolished *chrd* expression [47]. These observations also explain the necessity of the Wnt/Tcf pathway in *chrd* (or other organizer genes) activation [25,26,28,44,47,48]. The ChIP-PCR results validated the possibility of direct binding of Gsc and Tcf7 to the endogenous *chrd* promoter. However, how Gsc recognizes both GRE1 and WRE is an interesting subject for future investigation. It is also possible that Gsc and Tcf7 physically interact, and the complex of homeobox TFs collectively activates *chrd* transcription. We observed that Gsc requires intact GRE and WRE, and point mutations or deletions of WRE have completely lost Gsc activity. In contrast, the Tcf7 response remained within WRE only; a point mutation in GRE1 did not affect Tcf7 activity. This may be due to the heterodimer nature of the Gsc-Tcf7 complex, wherein Gsc interacts with GRE and Tcf7 binds to WRE. Similarly, it has been reported that Tcf(s) bind to unique WRE and interact with other co-activators (or co-repressors) to regulate the target gene expression [26]. This report indicates that Gsc-Tcf7 may interact to activate *chrd* transcription. However, further investigations are necessary to understand the detailed molecular mechanisms required.

**Figure 6:**
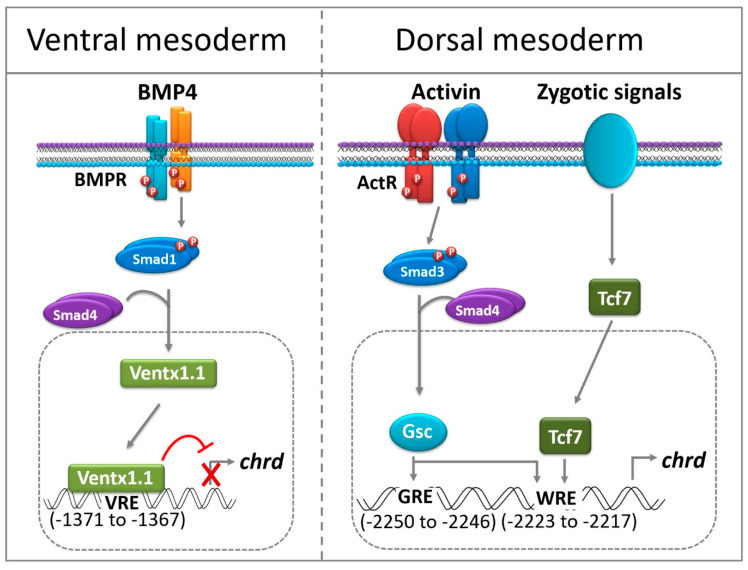
A putative model of Ventx1.1 and Gsc/Tcf7-mediated regulation of *chrd* transcription. A schematic representation of Ventx1.1-mediated negative regulation of *chrd* transcription by direct binding to VRE within the *chrd* promoter region during gastrula for ectoderm specification in *Xenopus* embryos. Whereas in the organizer (or dorsal mesoderm) region, Gsc interacts with GRE/WRE for activation. Tcf7 specifically binds to the WRE and activates *chrd* transcription.

In summary, *chrd* expression is tightly regulated spatiotemporally during embryonic development. Previously, we reported that Smad2 and Smad3 bind to ARE within the *chrd* promoter and positively regulate *chrd* transcription during gastrulation [16]. During neuroectoderm specification, the neural repressor Foxd4l1.1 inhibits *chrd* transcription to promote the neuronal program [17]. The present study demonstrated that the *chrd* promoter also contained functional VRE, GRE, and WRE (Figure 6).We have discussed earlier that Ventx1.1 interacts with VRE and blocks the transcriptional process. Gsc and Tcf7 bind to GRE and WRE to drive *chrd* activation. Collectively, our study provides evidence and an understanding of the unique *chrd* transcriptional regulatory mechanisms during *Xenopus* gastrulation (Figure 6).

## Figures and Tables

**Table 1:** Primers used for serially deleted *chrd(*-*2250)* promoter constructs.

Constructs	Primer Name	Sequences (5′ to 3′)
Upstream primer	Chrd (-2250)_F	GGGGCTAGCGAACGATACTTCAAGGACAAT
	Chrd (-2239)_F	GGGGCTAGCCAAGGACAATTGATAGAGAAAA
	Chrd (-2228)_F	GGGGCTAGCTGATAGAGAAAAGAAAGT
	Chrd (-2206)_F	GGGGCTAGCCCACTATCCCCACTAAGATGA
	Chrd (-2155)_F	GGGGCTAGCAGGCATACTTTGGTTTGTGTGT
	Chrd (-2135)_F	GGGGCTAGCGTATTCTGTGTAGCAAATCA
	Chrd (-2104)_F	GGGGCTAGCTGTTGCTTCTGTTTTCCACC
	Chrd (-2075)_F	GGGGCTAGCTGCAAGTCGAGATCATTGTGT
	Chrd (-1862)_F	GGGGCTAGCAAGAACACAGTGCCAGGCACT
	Chrd (-1473)_F	GGGGCTAGCCAGTAGGTTAGATGAACTACT
	Chrd (-790)_F	GGGGCTAGCACACTCTCTACCCCAATTCT
	Chrd (-386)_F	GGGGCTAGCCTTGACGGCTTTGTTTGCTT
	Chrd (-198)_F	GGGGCTAGCGTGTGGGTACAGAGCAACAA
Downstream primer	Chrd (-2250)_R	GGGCTCGAGTTTTGTGGTTCCAAACGTTCT

**Table 2:** Primers used for RT-PCR amplification of several sets of genes.

Gene	Primer Name	Sequences (5′ to 3′)	Cycles
*Chrd*	Chrd_F	TTAGAGAGGAGAGCAACTCGGGCAAT	25
	Chrd_R	GTGCTCCTGTTGCGAAACTCTACAGA	
*Gsc*	Gsc_F	GCTGATTCCACCAGTGCCTCACCAG	30
	Gsc_R	GGTCCTGTGCCTCCTCCTCCTCCTG	
*eGFP*	eGFP_F	GACGTAAACGGCCACAAGTT	32
	eGFP_R	CCTCCTTGAAGTCGATGCCC	
*Ventx1.1*	Ventx1.1_F	CCTTCAGCATGGTTCAACAG	28
	Ventx1.1_R	CATCCTTCTTCCTTGGCATCTCCT	
*ODC*	ODC_F	GTCAATGATGGAGTGTATGGATC	25
	ODC_R	TCCATTCCGCTCTCCTGAGCAC	

**Table 3:** Primers used for site-directed mutagenesis.

Mutated Sites	Primer Name	Sequences (5′ to 3′)	Cycles
GRE	*Chrd(-2250)mGRE_F*	ACGCGTGCTAGCGAGTAATACTTCAAGGACA	20
	*Chrd(-2250)mGRE_R*	TGTCCTTGAAGTATTACTCGCTAGCACGCGT	
WRE	*Chrd(-2250)mWRE_F*	GACAATTGATAGAGAGGAGAAAGTCTAT	20
	*Chrd(-2250)mWRE_R*	ATAGACTTTCTCCTCTCTATCAATTGTC	
VRE	*Chrd(-2250/-1473)mVRE_F*	TTCTTTCAGTTCCTAGGGGTTATTAATTACTTT	20
	*Chrd(-2250/-1473)mVRE_R*	AAAGTAATTAATAACCCCTAGGAACTGAAAGAA	

**Table 4:** Primers used for ChIP-PCR amplification.

Site	Primer Name	Sequences (5′ to 3′)	Cycles
ChIP-GRE	Chrd(GRE)_F	CGATACTTCAAGGACAATTG	25
	Chrd(GRE)_R	AGGTGGAAAACAGAAGCAAC	
ChIP-WRE	Chrd(WRE)_F	CGATACTTCAAGGACAATTG	25
	Chrd(WRE)_R	AGGTGGAAAACAGAAGCAAC	
ChIP-VRE	Chrd(VRE)_F	TCGGGTCTGGTACAGCAA	27
	Chrd(VRE)_R	ACCAGGAGAGGGAGATGT	
Internal negative control (C)	Control_F	TGCGCCGACTAAGTTTCCT	25
	Control_R	ATTAGTGACCCATGGCAGG	

## Data Availability

Original data are available on reasonable request from the corresponding authors.

## Supplementary Materials

The following supporting information can be downloaded at: https://www.mdpi.com/article/10.3390/cells12060874/s1. Figure S1: Serially deleted chrd promoter constructs injected with and without gsc mRNA at the one-cell stage. The relative luciferase activity was measured at stage 11; Figure S2: Identification of GRE1 within the chrd promoter. (A and B) The systematic representation of two targeted point mutations (namely GRE1 and GRE2, shown in the dotted box) was generated by site-directed mutagenesis in the upstream 11 bp (from -2250 to -2239) within the chrd promoter. (C) The mutated *chrd(-2250)mGRE1*, *chrd(-2250)mGRE2*, and wild-type *chrd(-2250)* promoter constructs were then injected with and without gsc mRNA at the one-cell stage. The relative luciferase activity was measured at stage 11.
