# Supplementary material for: Two Homeobox Transcription Factors, Goosecoid and Ventx1.1, Oppositely Regulate Chordin Transcription in Xenopus Gastrula Embryos
Source: Cells. 2023 Mar 11;12(6):874. doi: 10.3390/cells12060874 (PMC10047115; doi:10.3390/cells12060874)
Supplement: Supplementary file 1 [file cells-12-00874-s001.zip › cells-2197949-supplementary.pdf]

Two homeobox transcription factors, Goosecoid and Ventx1.1, oppositely regulate chordin transcription in *Xenopus* gastrula embryos

Vijay Kumar[1], Zobia Umair[1], Unjoo Lee[2*], and Jaebong Kim[1*]

[1]*Department of Biochemistry, Institute of Cell Differentiation and Aging, College of Medicine, Hallym University, Chuncheon, Gangwon-Do 24252, Republic of Korea*
[2]*Department of Electrical Engineering, Hallym University, Chuncheon, Gangwon-Do 24252, Republic of Korea*

*Address correspondence to:
Jaebong Kim
Fax: +82-33-244-8425;
Tel: +82-33-248-2544;
E-mail: jbkim@hallym.ac.kr

Unjoo Lee
Tel: +82-33-248-2354;
E-mail: ejlee@hallym.ac.kr

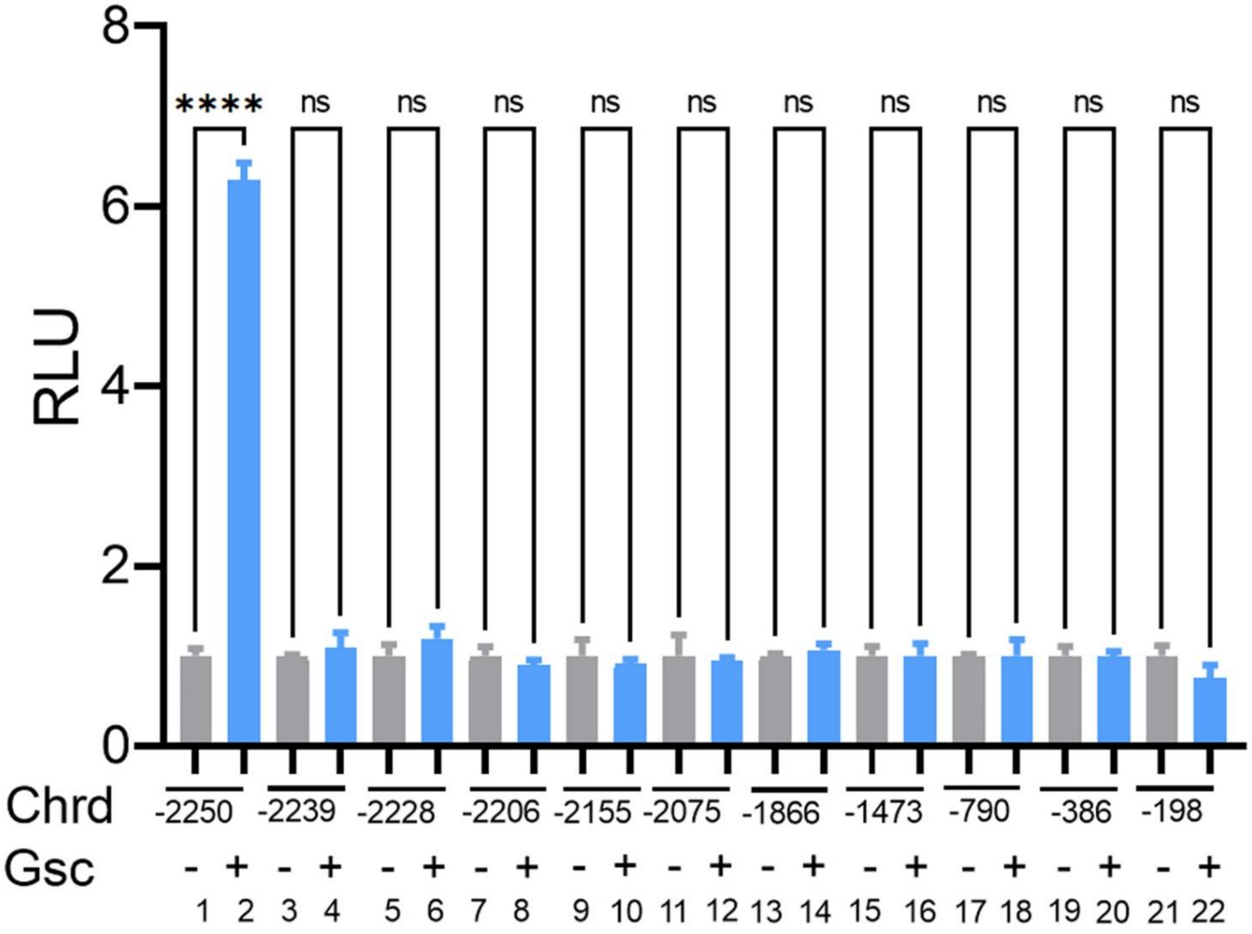

Figure S1. Serially deleted *chrd* promoter constructs injected with and without *gsc mRNA* at the one-cell stage. The relative luciferase activity was measured at stage 11.

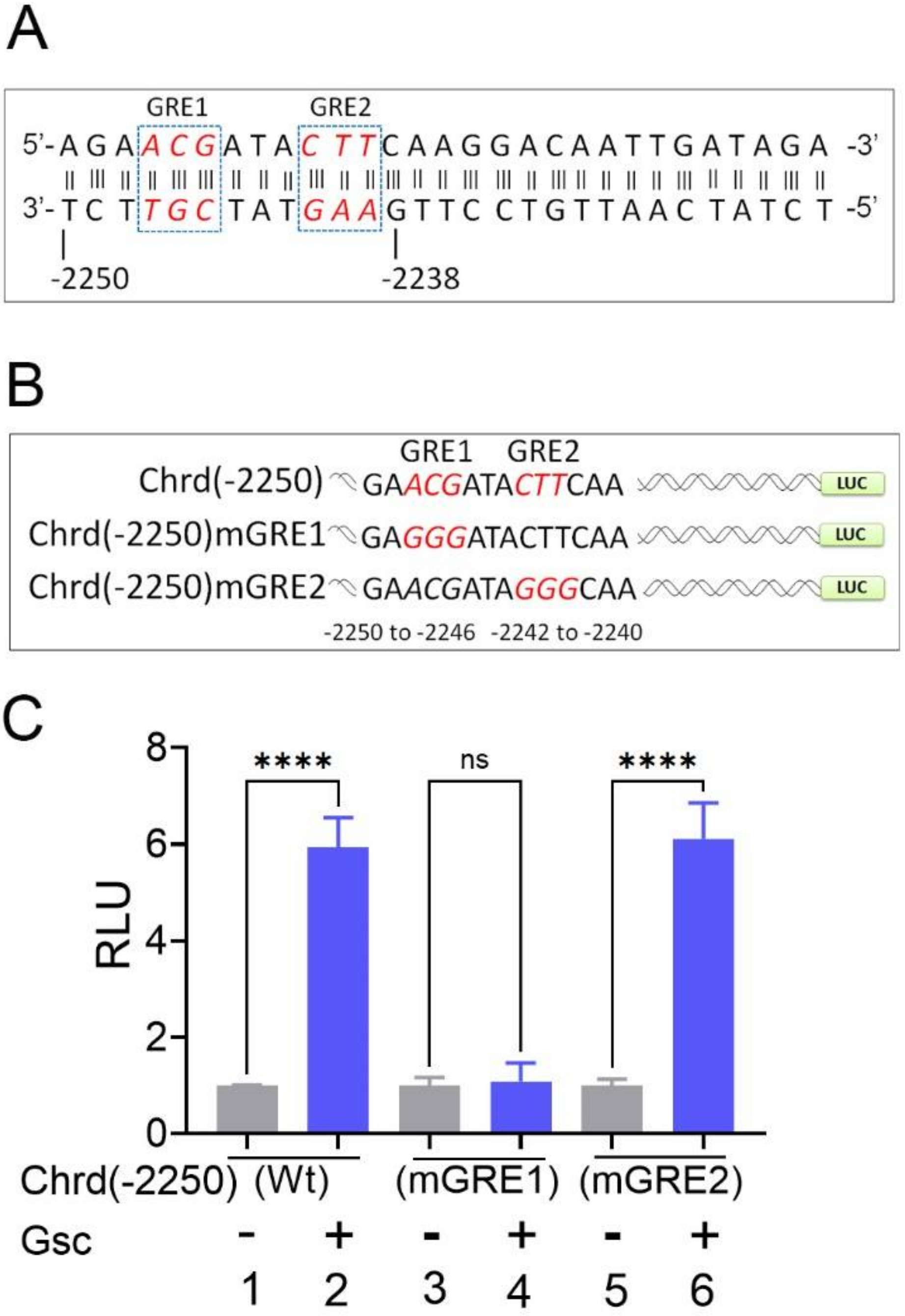

Figure S2. Identification of GRE1 within the *chrd* promoter. (A and B) The systematic representation of two targeted point mutations (namely GRE1 and GRE2, shown in the dotted box) was generated by site-directed mutagenesis in the upstream 11 bp (from -2250 to -2239) within the *chrd* promoter. **(C)** The mutated *chrd(-2250)mGRE1*, *chrd(-2250)mGRE2*, and wild-type *chrd(-2250)* promoter constructs were then injected with and without *gsc mRNA* at the one-cell stage. The relative luciferase activity was measured at stage 11.
